# Association between duration or intensity of tobacco smoking and olfactory function: A cross-sectional study among smokers willing to quit smoking

**DOI:** 10.18332/tid/211073

**Published:** 2025-12-20

**Authors:** Cornelia Wälchli, Valerie Grünig, Kali Tal, Nicolas Rodondi, Ivan Berlin, Jean-Paul Humair, Anja Frei, Susanne Pohle, Julian Jakob, Reto Auer, Sophia C. Poletti, Anna Schoeni

**Affiliations:** 1 Institute of Primary Health Care (BIHAM), University of Bern, Bern, Switzerland; 2 Department of General Internal Medicine, Inselspital, University Hospital Bern, Bern, Switzerland; 3 Department of Medical Pharmacology, Pitié-Salpêtrière University Hospital, Paris, France; 4 Primary Care Division, Geneva University Hospital, Geneva, Switzerland; 5 Epidemiology, Biostatistics and Prevention Institute, University of Zurich, Zurich, Switzerland; 6 Clinic for Pneumology and Sleep Medicine, HOCH Health Ostschweiz, Cantonal Hospital St. Gallen, St. Gallen, Switzerland; 7 Department of Pediatrics, Inselspital, University Hospital Bern, Bern, Switzerland; 8 Graduate School for Health Sciences, University of Bern, Bern, Switzerland; 9 Center for Primary Care and Public Health (Unisanté), Lausanne, Switzerland; 10 Department of Otorhinolaryngology, Head and Neck Surgery, Inselspital, University Hospital Bern, Bern, Switzerland

**Keywords:** smoking cessation, tobacco smoking, olfactory dysfunction, Burghart’s Sniffin Sticks

## Abstract

**INTRODUCTION:**

A range of studies suggests that people who smoke tobacco have impaired olfactory function, but few have explored the association between smoking history, such as duration or intensity, and olfactory function. We aimed to determine the prevalence of olfactory dysfunction among adult smokers and to test the association between duration or intensity of smoking and olfactory function.

**METHODS:**

For this cross-sectional study we consecutively invited adult smokers, participating in a smoking cessation trial conducted in five Swiss study sites, to undergo olfactory function testing at baseline from September 2020 to June 2021. We tested olfactory function with the Burghart’s Sniffin’ Sticks 16-item identification test resulting in an olfactory identification score (OIS) of 0–16 points. We defined olfactory dysfunction as an OIS ≤11 points. We fitted multivariable regression models to test the association between the OIS or olfactory dysfunction and self-reported smoking parameters [cigarettes per day (CPD), years of smoking (YOS) and pack-years] adjusted for relevant confounders such as demographics, substance use and comorbidities.

**RESULTS:**

Of 388 eligible participants, 375 (96.7%) completed the olfactory testing. Mean age was 39.0 years (SD=13.2), and 44.8% identified as women. The participants smoked on average 15 (SD=7.1) cigarettes per day for a median duration of 18 years (IQR: 11–28). Mean OIS was 13.3 (SD=1.8) and 12.0% had olfactory dysfunction. Olfactory dysfunction was significantly associated with pack-years (OR=1.03; 95% CI: 1.00–1.05) but not with YOS or CPD. OIS was negatively associated with pack-years (coefficient= -11.11; 95% CI: -4.29 – -17.94). OIS was not significantly associated with YOS or CPD.

**CONCLUSIONS:**

Among smokers smoking ≥5 cigarettes per day participating in a smoking cessation trial, about one in ten had olfactory dysfunction. Higher number of pack-years were associated with a worse measure of olfactory function and with olfactory dysfunction.

**CLINICAL TRIAL REGISTRATION:**

This sub-study of the ESTxENDS trial is pre-registered on the official website of ClinicalTrials.gov

**IDENTIFIER:**

NCT04617444

## INTRODUCTION

Olfactory dysfunction is characterized by the impaired ability to detect and distinguish odors^1-4^. The likelihood of olfactory dysfunction increases with age and is more common in men than women^5-10^. Impaired olfactory function can be dangerous, for example when people cannot detect the presence of smoke or potentially toxic substances^11^, and even under normal circumstances it can limit enjoyment of daily activities like cooking and eating, thus lowering quality of life^12^.

The potential causes of olfactory dysfunction include exposure to tobacco cigarette smoke^1,13-16^, which may disrupt the cycle of loss and regeneration of olfactory neurons^17-19^, increase the rate of apoptosis in the olfactory epithelium^17^, or reduce olfaction through chronic sinonasal inflammation and squamous metaplasia^20-23^. A systematic review and meta-analysis estimated the prevalence of olfactory dysfunction among persons who smoke to range from 4% to 25%^13^. However, the studies included mostly viewed smoking as a dichotomous factor or used pack-years as the indicator of one’s smoking history. This leaves the question open whether the association between tobacco smoking and olfactory dysfunction is dose-related, time-related or a combination of the two.

Of the studies retrieved in an epidemiological review, some used subjective measures of olfactory function, and these tend to find lower prevalence than studies based on objective olfactory assessments, perhaps because people think their sense of smell is better than it actually is^9,24^. Individuals who overestimate their sense of smell are also likely to underestimate their impairment or to believe their sense of smell has improved over time^9^. To increase the reliability of the prevalence of olfactory dysfunction, prevalence estimates should be based on validated olfactory function tests like the Burghart’s Sniffin’ Sticks 16-item Identification Test^25-29^.

We first determined the prevalence of olfactory dysfunction based on an objective olfactory function test in adult smokers in Switzerland participating in a smoking cessation trial. Secondly, we tested the association between their smoking history (years of smoking and cigarettes per day, alone and jointly measured as pack-years) and olfactory function.

## METHODS

### Study design and participants

Participants in this cross-sectional study were enrolled in the ESTxENDS trial (Efficacy, Safety and Toxicology of Electronic Nicotine Delivery Systems as an aid for smoking cessation: The ESTxENDS multicenter randomized controlled trial (registered in Clinical-Trials.gov with Identifier: NCT03589989)^30^. Participants were adult smokers (aged ≥18 years), who had smoked ≥5 cigarettes per day for the last 12 months before they enrolled; all were willing to quit smoking. The trial excluded pregnant and breastfeeding smokers, electronic nicotine delivery systems (ENDS) users, smokers who had used nicotine replacement therapy (NRT) in the three months prior to inclusion and people unable to understand the study processes. The trial was conducted in five Swiss cities: Bern, Zurich, Lausanne, Geneva, and St. Gallen. Participants were included in ESTxENDS from 16 July 2018 to 30 June 2021. Olfactory function assessments started on 9 September 2020, we therefore report on a restricted consecutive sample of ESTxENDS participants included from that date to 30 June 2021. The sample size for olfactory function assessments was determined by the sample size of the ESTxENDS randomized controlled trial and we did not compute a formal sample size when we planned these additional assessments.

### Measures

*Olfactory function*

We assessed olfactory function with the Burghart’s Sniffin’ Sticks 16-item Identification Test, first developed in 1997 and validated in several European countries^25-28,31^. The test contains 16 felt-tipped pens scented with orange, leather, cinnamon, peppermint, banana, lemon, licorice, turpentine, garlic, coffee, apple, clove, pineapple, rose, anise, or fish. After sniffing each stick, participants could choose one of four answers printed on multiple choice cards. Participants were asked to consume only water and not smoke or chew gum for 15 minutes before the test.

The pen cap was removed, and the pen placed about 2 cm in front of the participant’s nostrils for about three seconds. After sniffing, participants chose among four possible odors listed on the card. The sum of correct answers was the participant’s odor identification score (OIS); the highest possible score was 16 points.

We categorized participants with an OIS >11 points as having normal olfaction (normosmic) and ≤11 points as having olfactory dysfunction (hyposmic). The 11-point cutoff was based on normative data in over 3000 participants; normosmic was defined as an OIS over the 10th percentile in a healthy population^25^.

*Smoking history*

We assessed each participant’s smoking history with data collected via a questionnaire at the ESTxENDS baseline visit. We assessed smoking history in three different ways: 1) self-reported average number of cigarettes smoked per day (CPD) over the last 12 months; 2) number of years of smoking (YOS; we subtracted the length of time participants self-reported they had not smoked from their age at onset of regular smoking); and 3) pack-years (a pack comprises 20 cigarettes; calculated by dividing the number of cigarettes smoked per day (CPD) by 20 to obtain packs per day, then multiplying by YOS).

*Covariates*

We collected demographic data, cannabis, alcohol and illicit drug use and smoking characteristics at baseline. The demographic data were gender, age and current work situation. Participants completed the Alcohol Use Disorders Identification Test-Concise (AUDIT-C)^32^, which assesses alcohol consumption frequency, quantity, and instances of consuming ≥6 standard drinks. To identify potential problematic alcohol use, we used the standard cutoff scores of ≥3 points for women and ≥4 points for men. With respect to cannabis use, participants were asked whether they had used cannabis ≥3 times in the six months preceding their baseline visit. We assessed current illicit drug use by asking participants if they had used any illicit drug in the last 30 days prior to the baseline visit. Current drug use was defined as any use within the past 30 days. Substances considered illicit included LSD (lysergic acid diethylamide), mushrooms, phencyclidine, ketamine, (met)amphetamines, MDMA (3,4-methylenedioxymethamphetamine, commonly known as ecstasy), cocaine, heroin, morphine, methadone, codeine, and inhalants. Current drug use was treated as binary variable (yes, no) in the analysis. We used the 9-item Patient Health Questionnaire (PHQ-9)^33^ to assess self-reported depressive symptoms at baseline. Depression symptoms severity was categorized as no depression (<5 points), mild depression (5–9 points), and moderate to severe depression (>9 points).

### Statistical analysis

For descriptive statistics, we present categorical variables as frequencies and percentages, and continuous variables as mean with standard deviation (SD) or median with interquartile range (IQR), as appropriate. We then analyzed the association between smoking parameters (CPD, YOS, and pack-years) and the olfactory identification score (OIS) or olfactory dysfunction. We used univariable and multivariable logistic regression models to determine the association between smoking parameters and olfactory dysfunction, computing odds ratios. We also used univariable and multivariable linear regression models to examine the association between smoking parameters and OIS. Because olfactory identification scores were not distributed normally, included outliers, and failed to meet the assumptions of linearity and homoscedasticity, we applied a cubic transformation to the OIS to fulfill the assumptions of a linear regression^34^. We adjusted all models for gender, current work situation, alcohol consumption (AUDIT-C), cannabis consumption in the last 6 months, current illicit drug use, age, depression score (PHQ9), and study site. These variables were selected based on their biological plausibility as potential confounders of the association between tobacco smoking and olfactory function. Statistical analyses were performed in Stata Version 16 (StataCorp, College Station, USA). The level of statistical significance was set at 0.05, all tests were two-tailed.

## RESULTS

We invited 388 consecutive ESTxENDS participants to participate in olfactory testing; of those 375 (96.6%) participants completed the testing. Of the 375, 168 (45.4%) were women. Age ranged from 18 to 74 years with a mean age of 39.0 years, and 4.3% were aged >65 years. Additional participant characteristics are listed in Table 1.

**Table 1:** Participant characteristics from a cross-sectional study on tobacco smoking and olfactory function, Switzerland, September 2020–June 2021 (N=388)

*Characteristics*	*Total* *participants* *(N=388)* *n (%)*	*Completed* *olfactory testing* *(N=375)* *n (%)*
**Gender** (Women)	176 (45.36)	168 (44.80)
**Age** (years)		
mean (SD) >65	39.0 (13.3) 17 (4.4)	39.0 (13.2) 16 (4.3)
**Study site**		
Bern	73 (18.81)	67 (17.87)
Genève	88 (22.68)	86 (22.93)
Lausanne	23 (5.93)	19 (5.07)
St. Gallen	100 (25.77)	100 (26.67)
Zürich	104 (26.80)	103 (27.47)
**Work status**		
Employed/self-employed	278 (71.65)	268 (71.47)
Unemployed/in formation	110 (28.35)	107 (28.53)
Depression symptoms severity*		
None	224 (57.88)	217 (57.87)
Mild	112 (28.94)	108 (28.80)
Moderate/moderately severe	51 (13.18)	50 (13.33)
**Current hazardous alcohol consumption†**	189 (48.96)	183 (48.93)
Cannabis use ≥3 times in the last 6 months	93 (23.97)	91 (24.27)
Current illicit drug use	32 (8.25)	32 (8.53)
***Smoking history***	***Median (IQR)***	***Median (IQR)***
Years of smoking	18 (11–28.5)	18 (11–28)
Cigarettes per day	15 (10–20)	15 (10–20)
Pack-years	13.8 (6.6–25.4)	13.5 (6.5–24.0)

* Categories of depression symptoms based on the Patient Health Questionnaire-9 (PHQ9) score: no depression (<5 points), mild depression (5–9 points), and moderate to severe depression (>9 points).

† AUDIT-C (Alcohol Use Disorders Identification Test-Concise) score: ≥3 women and ≥4 men, defined as hazardous alcohol consumption. IQR: interquartile range.

### Smoking history

Years of smoking ranged 1–59 years, after we subtracted periods of no-smoking, with median of 18 years (IQR: 11–28). The median number of cigarettes smoked per day was 15 (IQR: 10–20), range 5–60 cigarettes per day. Median pack-years was 13.5 (IQR: 6.5–24.0); half of the participants smoked 1 pack or 20 cigarettes per day for 13.5 years.

### Olfactory function

**Figure 1:**
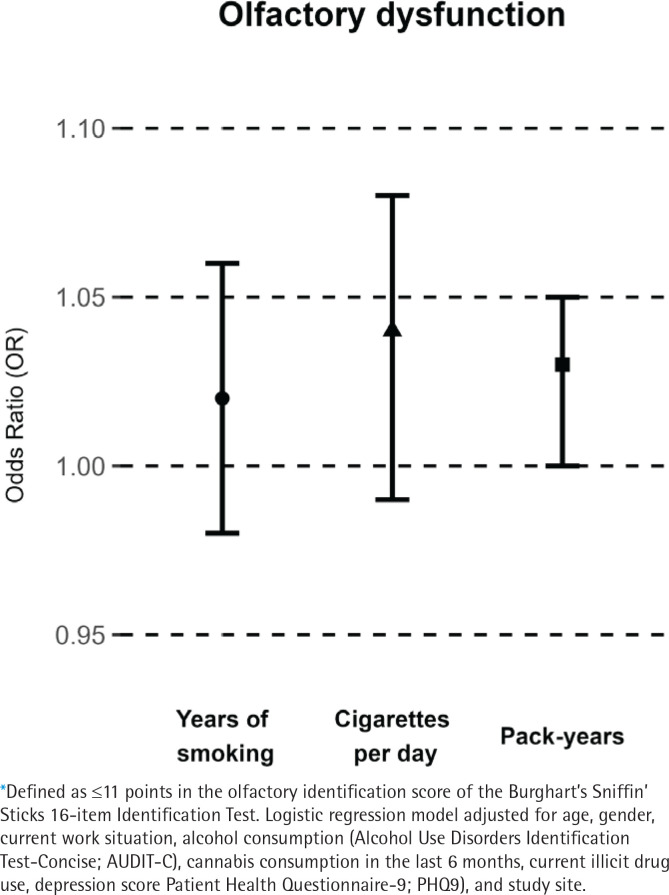
Association between smoking parameters and olfactory dysfunction* in participants who completed olfactory testing, a cross-sectional study, Switzerland, September 2020–June 2021 (N=375)

The overall prevalence of olfactory dysfunction was 12.0%; more men (15.0%) than women (8.3%) had an olfactory dysfunction. In multivariable adjusted logistic regression models, YOS (OR=1.02; 95% CI: 0.98–1.06) and number of CPD (OR=1.04; 95% CI: 0.99–1.08) were not significantly associated with olfactory dysfunction; pack-years (OR=1.03; 95% CI: 1.00–1.05) was significantly associated with olfactory dysfunction (Table 2). This is visualized in Figure 1. In gender-specific models, no statistically significant associations were observed between smoking parameters and olfactory dysfunction for YOS (men: OR =1.02; 95% CI: 0.97–1.06; women: OR=1.02; 95% CI: 0.95–1.10) or CPD (men: OR=1.03; 95% CI: 0.98–1.08; women: OR=1.11; 95% CI: 0.99–1.24). However, the gender-specific models for pack-years indicated a statistically significant association with olfactory dysfunction among women (OR=1.07; 95% CI: 1.01–1.13) but not among men (OR=1.02; 95% CI: 0.99–1.05).

**Table 2:** Association between smoking parameters and olfactory dysfunction* in participants who completed olfactory testing, a cross-sectional study, Switzerland, September 2020–June 2021 (N=375)

*Variables*	*OR (95% CI)*	AOR (95% CI)**
Years of smoking	**1.04 (1.02–1.07)**	1.02 (0.98–1.06)
Cigarettes per day	**1.06 (1.02–1.10)**	1.04 (0.99–1.08)
Pack-years	**1.03 (1.02–1.06)**	**1.03 (1.00–1.05)**

* Defined as ≤11 points in the olfactory identification score of the Burghart’s Sniffin’ Sticks 16-item Identification Test.

** AOR: adjusted odds ratio; logistic regression model adjusted for age, gender, current work situation, alcohol consumption (Alcohol Use Disorders Identification Test-Concise. AUDIT-C), cannabis consumption in the last 6 months, current illicit drug use, depression score Patient Health Questionnaire-9; PHQ9), and study site.

Mean OIS was 13.3 points (SD=1.8). In multivariable adjusted linear regression models, YOS (coefficient= -9.48; 95% CI: -19.62–0.66) and number of CPD (coefficient= -11.74; 95% CI: -23.74–0.26) were not associated with OIS; pack-years (coefficient= -11.11; 95% CI: -17.94 – -4.29) were significantly associated with OIS (Table 3). The direction of the association between pack-years and olfactory identification score was negative, indicating that greater smoking exposure was associated with poorer olfactory performance. The model including pack-years had an adjusted R^2^ of 0.106 (95% CI: 0.029–0.183), compared with 0.083 (95% CI: 0.013–0.153) when pack-years was excluded, corresponding to an additional 2.3% of variance explained.

**Table 3:** Association between smoking parameters and olfactory identification score* in participants who completed olfactory testing, a cross-sectional study, Switzerland, September 2020–June 2021 (N=375)

*Variables*	*Crude coeff (95% CI)*	*Adjusted coeff (95%* CI)**
Years of smoking	**-9.76 (-16.35–-3.18)**	-9.48 (-19.62–0.66)
Cigarettes per day	**-14.50 (-26.36–-2.64)**	-11.74 (-23.74–0.26)
Pack-years	**-10.32 (-15.69–-4.96)**	**-11.11 (-17.94 – -4.29)**

Coeff: coefficient.

* Olfactory identification score (0–16 points) of the Burghart’s Sniffin’ Sticks 16-item Identification Test was cubic transformed.

** Linear regression model adjusted for age, gender, current work situation, alcohol consumption (Alcohol Use Disorders Identification Test-Concise; AUDIT-C), cannabis consumption in the last 6 months, current illicit drug use, depression score Patient Health Questionnaire-9; PHQ9), and study site.

## DISCUSSION

In our study of adult smokers willing to quit, participating in an ongoing smoking cessation trial, prevalence of olfactory dysfunction was 12.0%. We explored the relationships between smoking parameters, e.g. daily smoked cigarettes (CPD), years of smoking (YOS), and pack-years, and both olfactory identification score (OIS) and olfactory dysfunction. We found no significant association with daily smoked cigarettes or years of smoking. Nevertheless, higher smoking exposure as measured in pack-years, was significantly associated with poorer odor identification and with increased odds of olfactory dysfunction.

Prevalence of olfactory dysfunction was 12.0% among participants in the ESTxENDS trial, whose mean age was 39.0 years and average number of cigarettes smoked per day was 15. We believe the mean age of participants in our sample largely explains the higher prevalence of olfactory dysfunction found in other studies^3,4,9^. In a large cross-sectional population-based study in Germany, including over a thousand participants with a mean age in the early fifties, the reported rates of olfactory dysfunction were higher and increased with smoking intensity to nearly every forth participant^1^. A population-based study in the US found olfactory dysfunction to vary mostly by age in the nearly 25000 participants included. It was found relatively uncommon among middle-aged adults and became more prevalent as the age increased, reaching its maximum with nearly two-thirds in people aged ≥80 years^9^.

Although men showed a higher overall prevalence of olfactory dysfunction, smoking exposure, measured as pack-years, was significantly associated with dysfunction only in women. Estrogen may contribute to sex differences, as it plays a crucial role in the development and maintenance of olfactory neurons^35^, potentially providing women with some biological protection. This protective effect may diminish with age, consistent with findings that gender differences in olfactory function are reduced in older adults^10^. However, our sample included only a limited number of older participants, which prevents further testing of this hypothesis among current smokers; we hope future studies will continue to explore these mechanisms. Taken together, our results indicate that cumulative smoking exposure interacts with sex-specific biological factors, and that behavioral and environmental factors may also contribute, thus highlighting the complex mechanisms underlying gender differences in olfactory dysfunction.

Our study identified a significant negative association between pack-years and OIS, suggesting that an increase in the cumulative smoking exposure correlates with worse OIS scores. Our findings align with those of Frye et al.^14^ who identified a negative correlation between cumulative smoking history, measured in pack-years, and University of Pennsylvania Smell Identification Test scores. They also align with the findings of Katotomichaelakis et. al.^16^, who reported a significant negative relationship between pack-years and OIS (also using Burghart’s Sniffin’ Sticks). In contrast to the study of Vennemann et al.^1^, we found no significant association between number of cigarettes smoked per day and olfactory dysfunction, but this might be due to the selected sample of only smokers smoking ≥5 cigarettes per day, with 15 smoked on average. While we did not find that smoking duration was significantly associated with olfactory dysfunction, we found olfactory dysfunction to be significantly associated with pack-years of smoking, which combines the information from years of smoking and cigarettes per day in one variable. This suggests pack-years can overcome potential misclassification of the exposure to tobacco smoking if using only information about only cigarettes per day or years of smoking in the models. For example, the variable years of smoking considers a person having smoked 1 cigarette per day over 10 years as equivalent to a person having smoked 40 cigarettes per day over 10 years, which represents a 40-fold difference in exposure. Misclassification of the exposure can drive the test of the null hypothesis of no differences between contrasts towards the null. In ESTxENDS, we collected extensive data on past exposure to compute pack-years of smoking and recommend future studies on the topic to engage in such extensive data collection to test the association between past exposure to tobacco smoking and olfactory function. Brämerson et al.^2^ performed such data collection on pack-years. Contrary to our findings and those of similar studies, they did not find that risk of olfactory dysfunction significantly increased with pack-years^14,16^. The authors suggested a substance-specific effect: some substances in tobacco smoke may affect olfaction more than others. If true, that could account for variation in study outcomes, if researchers tested different types and numbers of substances. These discrepancies suggest smoking may have a complex effect on olfaction, depending not just on duration and frequency, but also upon the components of tobacco smoke to which individuals are exposed or other factors.

### Limitations

Our study has four main limitations. First, we retrieved prevalence of olfactory dysfunction within a group of smokers willing to quit smoking and participate in a randomized controlled trial testing e-cigarettes for smoking cessation in Switzerland. Interpretation is thus limited to this population and prevalence might differ in other settings. Second, we relied on self-reported smoking history, which may have introduced non-differential misclassification of exposure, potentially biasing our estimates toward the null. It should be noted, however, that in daily clinical care, clinicians also rely on self-reported smoking history, so any imprecision in our estimates mirrors routine practice. Third, although we adjusted for several relevant covariates, the possibility of residual confounding cannot be excluded, as not all potential confounding factors could be accounted for. Finally, given the cross-sectional design, we can only describe associations and cannot infer that smoking history is causally related to olfactory outcomes; we cannot test and therefore exclude the possibility of reverse causality (e.g. impaired olfactory function influencing smoking behavior).

Further research is warranted to examine the association between olfactory dysfunction and smoking abstinence rates to determine if olfactory function changes after smoking cessation and if changes in olfactory function impacts smoking cessation rates.

## CONCLUSIONS

Among the participants in a smoking cessation trial, who smoked ≥5 cigarettes per day and were motivated to quit smoking, about one in ten had olfactory dysfunction at baseline. In men the percentage was nearly twice that in women. Olfactory outcome measures were negatively associated with pack-years but not with cigarettes smoked per day or years of smoking as a stand-alone factor, supporting the argument that intensity of smoking could harm olfaction via a combined increase in dosage and duration increase.

## Data Availability

The data supporting this research are available from the authors on reasonable request.
